# Design and Implementation of a Deep Learning System to Analyze Bovine Sperm Morphology

**DOI:** 10.3390/vetsci12101015

**Published:** 2025-10-21

**Authors:** Francisco Sevilla, Ignacio Araya-Zúñiga, Abel Méndez-Porras, Jorge Alfaro-Velasco, Efren Jiménez-Delgado, Miguel A. Silvestre, Rafael Molina-Montero, Eduardo R. S. Roldan, Anthony Valverde

**Affiliations:** 1 Instituto Tecnológico de Costa Rica, Universidad Nacional, Universidad Estatal a Distancia, Alajuela 223-21002, Costa Rica; f.sevilla@itcr.ac.cr; 2 Laboratory of Animal Reproduction, Research and Development Center for Sustainable Agriculture in the Humid Tropics, School of Agronomy, Costa Rica Institute of Technology, San Carlos Campus, Alajuela 223-21002, Costa Rica; igaraya@estudiantec.cr (I.A.-Z.); rafmolina@itcr.ac.cr (R.M.-M.); anvalverde@itcr.ac.cr (A.V.); 3 Computer Engineering Department, Costa Rica Institute of Technology, San Carlos Campus, Alajuela 223-21002, Costa Rica; amendez@itcr.ac.cr (A.M.-P.); joalfaro@itcr.ac.cr (J.A.-V.); efjimenez@itcr.ac.cr (E.J.-D.); 4 Functional Biology and Physical Anthropology, School of Cellular Biology, Burjassot Campus, Valencia University, 46100 Burjassot, Spain; miguel.silvestre@uv.es; 5 Agricultural Production Program, School of Agronomy, Costa Rica Institute of Technology, San Carlos Campus, Alajuela 223-21002, Costa Rica; 6 Department of Biodiversity and Evolutionary Biology, Natural Museum of Natural Sciences (CSIC), 28006 Madrid, Spain

**Keywords:** bovine sperm morphology analysis, image analysis, bovine reproduction, deep learning, YOLOv7

## Abstract

**Simple Summary:**

Sperm morphology analysis is important for diagnosing reproductive potential in bulls. Current visual evaluation, however, requires standardization and automation. For this reason, this work designed and implemented a computer-aided system to analyze bovine sperm morphology. We used the YOLOv7 object detection framework. Results indicate a balanced tradeoff between accuracy and efficiency. This alternative enhances efficiency and accuracy in animal reproduction laboratories in the field of veterinary reproduction.

**Abstract:**

Sperm morphology analysis is critical for assessing bovine fertility, since it provides insight into bull reproductive potential as well as subfertility and infertility. Traditional sperm morphology analysis is time-consuming, subjective, and prone to human error, all of which highlight the need for automated, objective solutions. This study presents the design and implementation of a computer-aided system for bovine sperm morphology analysis, leveraging deep learning models to detect and classify sperm cells based on their morphological characteristics. Using micrographs of bull sperm, we present a sequential deep learning framework that automatically detects morphological sperm aberrations. The model segments and analyzes each cell, identifying defects in the head, neck/midpiece, tail, and residual cytoplasm. Specifically, the system employs the YOLOv7 object detection framework, trained on a dataset of 277 annotated images comprising six morphological categories, to automatically identify and classify sperm abnormalities. The experimental results demonstrate a global mAP@50 of 0.73, precision of 0.75, and recall of 0.71, indicating a balanced tradeoff between accuracy and efficiency. By reducing reliance on manual analysis, this work enhances efficiency and accuracy in animal reproduction laboratories, contributing to veterinary reproduction through a cost-effective and scalable solution for sperm quality assessment.

## 1. Introduction

Bull breeding soundness evaluation (BSE) is the primary criterion for determining potential fertility under farm conditions [1,2,3]. Semen quality is a key indicator of reproductive function [4,5]; it helps identify infertility and its causes, ensures male reproductive soundness, and guides farm management strategies [6]. Sperm morphology analysis provides valuable insights into structural integrity and potential anomalies, proving a reliable predictor of male fertility [7], since sperm morphological abnormalities correlate with infertility [8,9,10].

The World Health Organization (WHO) has identified sperm morphology as crucial for evaluating sperm quality [11]. The standard protocol involves drying, staining, and assessing the cells, although fixation and dye-free approaches that immobilize spermatozoa through controlled pressure and temperature can also be employed [12]. The WHO recommends that, for each sample, a minimum of 200 spermatozoa are classified into five categories: normal, head defects, neck/midpiece defects (bent or broken), tail defects, and excess residual cytoplasm [13]. Manual sperm morphology assessments typically use microscopic observation, which is not only time consuming but also subject to significant inter-observer variability and human error [14].

The classification of sperm abnormalities in animals has used the idea of compensable or uncompensable abnormalities [15]. Compensable defects characterize sperm that cannot migrate or interact with the oocyte, preventing fertilization or the block to polyspermy. There would be little effect on fertility if insemination is carried out with sufficient numbers of competent sperm. In contrast, uncompensable defects permit sperm transport, oocyte penetration, and activation but compromise embryo viability and development, causing early embryonic loss or aberrant development; fertility would not be improved by increasing sperm numbers [16].

Automated systems that leverage advanced technologies such as deep learning have emerged as promising alternatives to manual evaluation [17]. These systems offer increased efficiency, reduced analysis time, and greater consistency [18]. Specifically, recent implementations of deep learning models, such as YOLO (You Only Look Once) [19,20,21], have demonstrated significant advantages in detecting human sperm morphological anomalies accurately and rapidly [22,23].

Artificial intelligence (AI), particularly convolutional neural networks (CNNs), has significantly transformed sperm morphology analysis across human and animal domains [24]. The lightweight YOLOv5 model, optimized for real-time human sperm detection, provides high accuracy with reduced computational complexity [25]. Other studies of different YOLO models highlight YOLOv5’s effectiveness in differentiating sperm from impurities [26]; they similarly note the CNN-based approach inspired by VGG architectures for automated assessment of human sperm morphology [27]. These authors created the modified human sperm morphology analysis (MHSMA) dataset, comprising 1540 non-stained, low-resolution images. This method addressed class imbalance through effective data augmentation. It achieved high F0.5 scores for detecting abnormalities in the acrosome (84.74%), head (83.86%), and vacuoles (94.65%), demonstrating suitability for clinical use due to its real-time capabilities on standard computational hardware.

The development of automated sperm morphology analysis systems depends significantly on advanced hardware and software integration. High-resolution microscopes, such as the Optika B-383Phi, (Optika^®^, Ponteranica, Italy) coupled with advanced imaging and labeling software like Roboflow, facilitate the accurate capture and annotation of sperm images. Furthermore, portable sperm analysis devices integrate smartphone-based imaging systems with deep learning models (MobileNet), underscoring the growing role of accessible and portable technologies in both clinical and veterinary environments [28].

This study proposes a deep learning-based automated system for analyzing bovine sperm morphology to streamline laboratory workflows, enhance accuracy, and provide reproducible assessments that integrate effectively into broader reproductive evaluation protocols. By automating critical aspects of sperm morphology analysis, our research transcends current limitations of traditional analyses and sets the stage for more robust and scalable fertility assessment practices.

## 2. Materials and Methods

### 2.1. Ethics Statement

This study used bull sperm samples obtained in collaboration with the Agricultural Production Program of the Costa Rica Institute of Technology. It was conducted in accordance with the laws and regulations for live animal experimentation in Costa Rica. Throughout this study, animals were handled to ensure their well-being and avoid unnecessary stress. This study adhered to ethical research principles, including the three Rs, and all procedures occurred in strict accordance with relevant guidelines and regulations to ensure ethical integrity and animal welfare. This research was approved by the Committee of the Center for Research and Development of Sustainable Agriculture for the Humid Tropics of the Costa Rica Institute of Technology in session 20/2023 Article 1.0, DAGSC-188-2023 and CIE-206-2023. The study also adhered to the ARRIVE guidelines (https://arriveguidelines.org/; accessed on 3 February 2025).

### 2.2. Semen Collection and Processing

We analyzed sperm morphology of sexually active Brahman bulls over 24 months of age with at least a 32 cm scrotal circumference (minimum parameter at 24 months for suitable breeding bulls in Costa Rica). The study also used bull semen from six bulls with varying morphologies, with at least two samples from each animal, totaling 12 ejaculates. These collections were carried out with at least 15 days between each.

We used a hydraulic press (Priefert^®^, model S04) to immobilize animals and the electroejaculation technique under BSE settings for ejaculate collection as described in [29]. Then, rectal feces were evacuated, and a 1–2 min rectal massage stimulated the accessory glands (vesicular glands and ampullae of the deferent ducts) and prostate, enhancing sexual response and relaxing the anal sphincter. A 75 mm diameter transrectal probe (Pulsator V, Lane Manufacturing, Denver, CO, USA) with a preprogrammed cycle, applied increasingly intense stimuli until ejaculation. Each bull underwent up to two cycles of 36 electrical stimuli, starting with six at 1–2 V, nine at 3–5 V, twelve at 6 V, and nine at 7 V, each lasting 3.5 s, concurrent with the rectal massage. Semen was collected directly from the penis using a sterile collection bag.

After collection, an aliquot of semen:extender was prepared in a 1:1 ratio (*v*/*v*) with Optixcell^®^ (IMV Technologies, L’Aigle, France) in Eppendorf^®^ tubes prewarmed at 37 °C to avoid temperature shock. Semen samples arrived at the laboratory in less than an hour. In the laboratory, they were further diluted at a 1:20 ratio, achieving an average concentration of 17.5–27.5 (×10^6^/mL) in 2 mL Eppendorf^®^ tubes. Both the semen and diluent were maintained at 37 °C. Sperm morphology was analyzed soon after samples arrived in the laboratory.

### 2.3. Sperm Morphology Analysis and Image Capture

During each bovine semen sample analysis, 10 μL was taken from each sample previously diluted in the range of 17.5–27.5 × 10^6^/mL and they were placed on a slide (75 × 25 × 1 mm), topped with a coverslip (22 × 22 mm), and the preparation placed in the Trumorph^®^ system (Proiser R+D, S.L., Paterna, Spain) for fixation, which subjected the sample briefly to 60 °C and a pressure of 6 kp enabling dye-free, pressure and temperature fixation for morphology evaluation [12]. The spermatozoa were thus fixed for evaluation under a B-383Phi microscope (Optika, Italy) with the 1× eyepiece and the 40× negative phase contrast objective, to evaluate their morphology.

**Figure 1:**
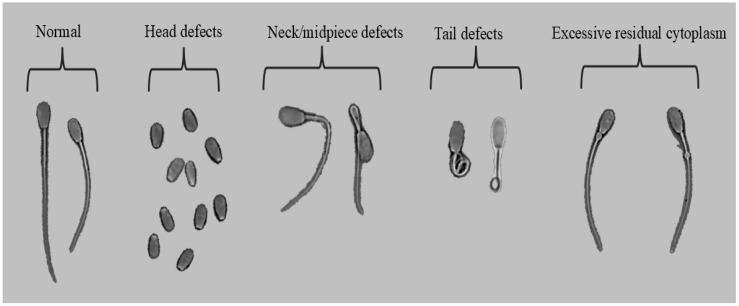
Examples of sperm morphology categories.

Sperm morphology were classified according with five categories: normal, head defects, neck/midpiece defects, tail defects, and excessive residual cytoplasm (Figure 1). Images were captured with the PROVIEW application (Optika, Ponteranica, Italy). Each image was labeled and stored in jpg format.

### 2.4. Dataset Preprocessing

**Figure 2:**
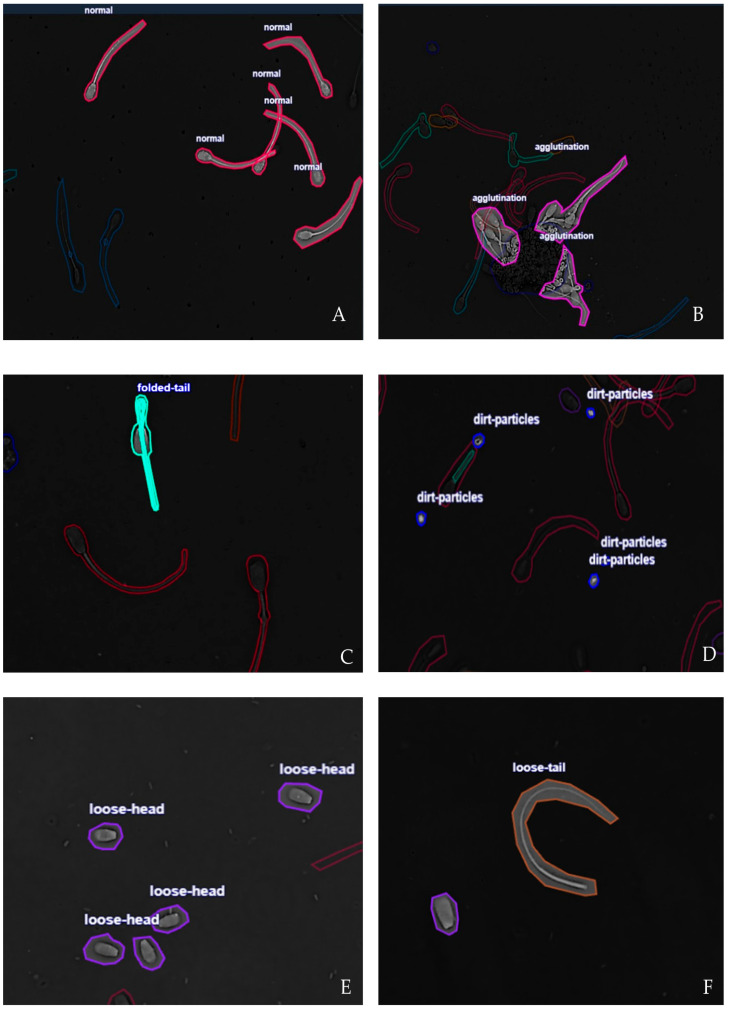
Representative annotated samples of the six morphological categories. (**A**) Normal, (**B**) Agglutination, (**C**) Folded tail (**D**) Dirt particles, (**E**) Loose head, and (**F**) Loose tail.

The samples were acquired using bright-field microscopy under standardized laboratory conditions to minimize variability in contrast and illumination. Six clinically relevant morphological categories reflect bovine sperm quality [30]. (1) Normal: spermatozoa with intact head, midpiece and tail morphology, without visible structural defects; these cells represent normal spermatogenesis and fertilization. (2) Agglutination: sperm cells adhered to each other by heads or tails, forming clusters. This condition interferes with progressive motility, reduces fertilization probability, and may reflect immunological or pathological conditions. (3) Dirt particles: noncellular debris or extraneous material in the microscopic field. Although not biological, such particles can be misclassified as sperm structures by automated systems, reducing detection accuracy. (4) Folded tail: abnormalities of the flagellum characterized by folding, coiling, or sharp bending; this sperm defects compromise motility by altering progressive movement and energy transfer. (5) Loose head: sperm heads detached from the midpiece and tail; this anomaly relates to structural fragility, incomplete spermatogenesis, or post-collection damage. It indicates reduced fertilization capacity and (6) Loose tail: sperm tails without their corresponding heads. This condition is associated with degenerative processes or physical damage, reflecting unstable cytoskeletal structures. Representative annotated samples for each morphological class are shown in Figure 2. The annotations were performed manually by trained specialists and subsequently validated by a veterinarian andrology expert to ensure label reliability.

### 2.5. Deep-Learning Model

We adopted the YOLOv7 architecture due to its balance between accuracy and inference speed in biomedical imaging [31]. YOLOv7 operates as a single stage object detector, predicting bounding boxes and class probabilities in a single forward pass, making it suitable for real-time laboratory applications. The model was pre-trained with Common Objects in Context (COCO) weights and fine-tuned on the bovine sperm dataset. This transfer learning strategy improved convergence and performance with limited annotated data [32].

### 2.6. Implementation and Training Configurations

The experiments were carried out on a workstation equipped with an Intel Xeon Silver 4310 CPU (Intel, Santa Clara, CA, USA) (2.10 GHz), 128 GB RAM, and an NVIDIA RTX 4090 GPU (NVIDIA Corporate, Santa Clara, CA, USA) (24 GB memory). The operating system was Ubuntu 22.04 LTS, with drivers and the CUDA toolkit version 12.1.

We further used the PyTorch 2.1 framework, with TorchVision 0.16. Training was accelerated with the cuDNN 8.9 backend. Data preprocessing, enhancement, and visualization were performed with the OpenCV and Albumentations libraries. The key hyperparameters were as follows: 200 training epochs, a batch size of 26, an initial learning rate of 1 × 10^−4^, and the Adam optimizer with default values β = (0.9, 0.999). A cosine annealing scheduler was applied to dynamically adjust the learning rate.

To ensure reproducibility, random seeds were fixed for the PyTorch (2.8.0), NumPy (2.3.0), and Python (3.13.6) standard libraries. All experiments were carried out under controlled laboratory conditions, and the complete codebase with configuration files is available on request. While the previous subsection described the computational environment and hyperparameters, this subsection focuses on the training workflow. For example, online data augmentation was applied during training, including random horizontal flipping, small affine transformations, brightness contrast adjustments, and Gaussian noise injection. These transformations increased data variability and simulated realistic laboratory imaging conditions.

Model optimization followed a transfer learning scheme: The pre-trained YOLOv7 weights in COCO were fine-tuned for the bovine sperm data. The cosine annealing scheduler gradually reduced the learning rate, promoting stable convergence. This strategy ensured that the model was efficiently adapted to the domain-specific morphology of bovine sperm cells while maintaining generalizability between categories.

Model performance was evaluated using four metrics commonly adopted in object detection and sperm morphology studies. In this binary classification framework for sperm morphology, the positive class is normal spermatozoa, and the negative class is abnormal spermatozoa. True positives (TP) are normal cells correctly identified; false positives (FP) are abnormal cells misclassified as normal; true negatives (TN) are abnormal cells correctly identified; and false negatives (FN) are normal cells misclassified as abnormal. The metrics used were: (1) Mean average precision at intersection over union (IoU) 0.5 (mAP@50) represents detection accuracy (Accuracy = [TP + TN]/[TP + TN + FP + FN]) measured at the 0.5 IoU threshold, as defined by the PASCAL VOC protocol [33]. (2) Precision (Precision = TP/[TP + FP]) is the proportion of correctly predicted positive detections among all predicted detections, indicating the rate of false positive control. (3) Recall (Recall = TP/[TP + FN]) represents the proportion of correctly predicted positive detections among all ground truth instances, indicating the rate of false negative control. (4) F1 score (F1 score = [2 * Precision * Recall]/[Precision + Recall]), in sperm-image classification, summarizes model performance by balancing precision (correct positive predictions out of all predicted positives) and recall (correct positive predictions out of all actual positives). These metrics enjoy established use in computer vision benchmarks and application in biomedical image analysis [34,35].

## 3. Results

YOLOv7 bovine sperm morphology classification revealed balanced performance in accuracy, robustness, and computational efficiency. The results are presented in terms of detection accuracy, computational efficiency, convergence analysis, and class level tradeoffs.

### 3.1. Experimental Results

Model performance detection accuracy was quantified using standard object detection metrics, including mAP@50, precision, recall, and F1 score. The system achieved a global mAP@50 of 0.73, a precision of 0.75, a recall of 0.71, and an F1 score of 0.73. The class level analysis indicated variability between the six categories, with normal spermatozoa and folded tail achieving higher precision and recall, while the loose tail class exhibited lower recall (Table 1).

Training and validation losses showed a consistent downward trend in all epochs, with no evidence of overfitting. Both the mean average precision (mAP) and F1 score stabilized after approximately 50 epochs, suggesting stable convergence of the optimization process.

For class-level performance and tradeoffs the results demonstrated that the morphological categories with well-defined structural features, such as normal and folded tail, achieved the highest scores. In contrast, categories with detached or fragmented structures, such as loose tail, presented lower recall, reflecting the difficulty in distinguishing them from background noise or other defects. These findings highlight dataset diversity and the balanced class representation in improving generalizability for minority or visually ambiguous classes. Although overall metrics indicate reliable detection, the tradeoff between precision and recall across categories suggests that further refinement may be required for deployment in diagnostic workflows (Table 2).

### 3.2. Experimental Conclusions

A closer inspection of the training dynamics revealed that precision exhibited smaller fluctuations after the first 30 epochs, suggesting that the model rapidly learned to minimize false positive predictions. Recall, on the other hand, improved more gradually, reflecting the difficulty in capturing all minority or visually ambiguous categories, such as loose tail and loose head sperm. Furthermore, the joint stability of both metrics indicates that the optimization process avoided overfitting to dominant classes while maintaining generalizability to underrepresented ones. In general, these findings confirm that the adopted training strategy produced a reliable compromise between false alarms and missed detections, crucial to ensuring robustness of automated morphology analysis under real laboratory conditions.

**Figure 3:**
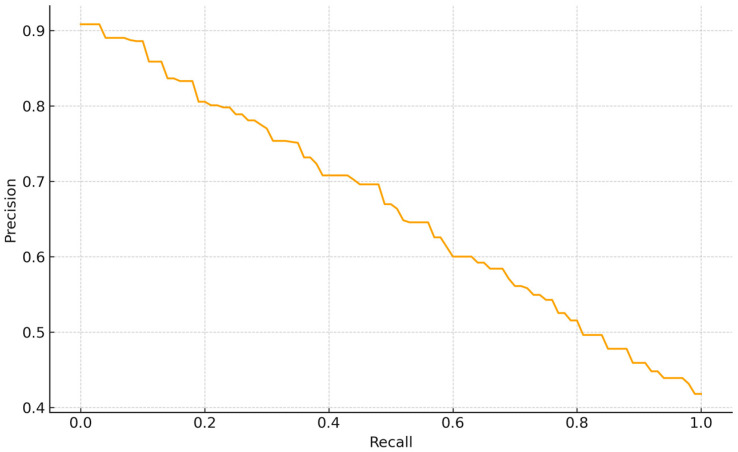
Precision–Recall curve YOLOv7 architecture for morphologically normal bull spermatozoa.

Despite the overall balanced performance, specific failure cases were identified during the evaluation. The Precision—Recall curve for the normal class detection shows a smooth tradeoff, indicating stable score calibration without abrupt collapses. Precision is 0.90 at very low recall (<0.05), 0.80 around R~0.20, 0.65 at R~0.60, and declines to ~0.50–0.55 at R~0.80, reaching ~0.42 at R~1.0. Thus, conservative thresholds yield very high precision at the cost of coverage, whereas operating the model at R~0.60–0.70 maintains P~0.60–0.65, giving an F1~0.62 (2PR/(P + R)). The absence of a high-precision plateau suggests that “normal” instances are moderately separable from non-normal classes but still include borderline cases (e.g., low contrast or partial overlaps). For Backbone-based supervised embedding (BBSE) style workflows, a practical operating point is R~0.60–0.70 (Figure 3).

**Figure 4:**
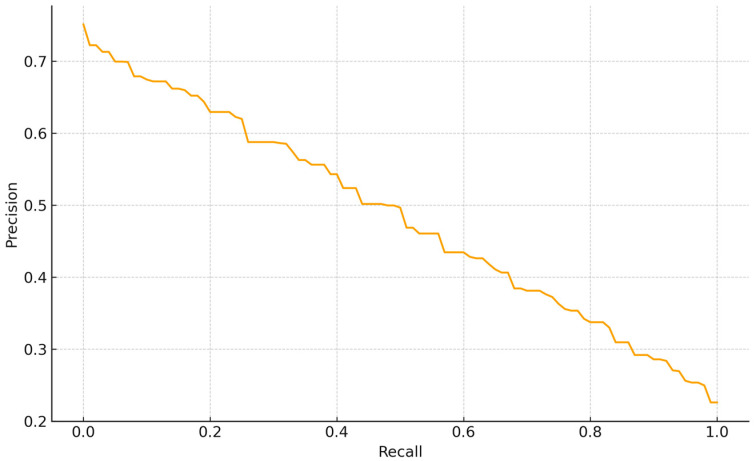
Precision—Recall curve YOLOv7 architecture for bull sperm tail abnormalities.

The PR curve for tail abnormalities exhibits a smooth, nearly linear tradeoff without a high-precision plateau, indicating moderate separability of this class. Precision is ~0.73 at very low recall (<0.05), declines to ~0.50 at R~0.50 (F1~0.50), reaches ~0.35–0.40 by R~0.70–0.75, and approaches ~0.23 as R~1.0. Thus, conservative thresholds achieve *p* ≥ 0.65 only at low recall (R ≤ 0.15). For BBSE workflows, a balanced operating point around R~0.55–0.65 (P~0.45–0.55) offers a reasonable compromise between coverage and review burden, consistent with this class contributing less to overall average precision than better-delimited categories (Figure 4).

**Figure 5:**
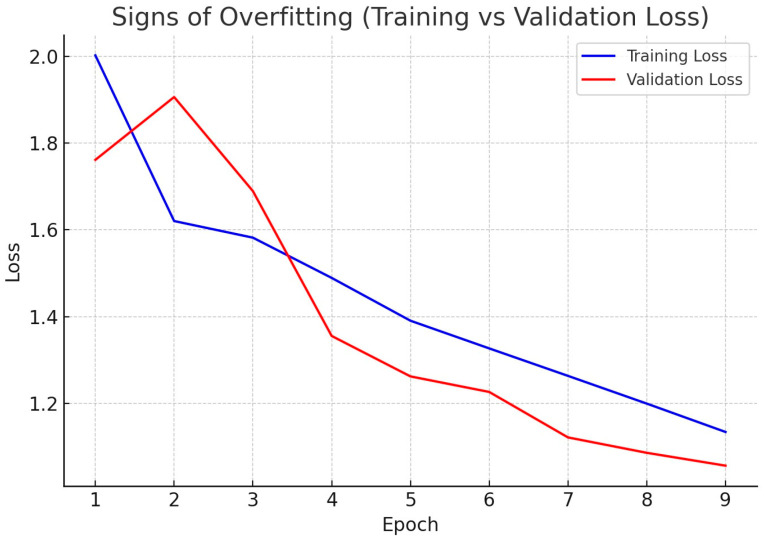
Training and validation loss during YOLOv7 training for bovine sperm morphology detection. Blue line (training loss) represents the model’s error on the training data. As shown, it decreases steadily across epochs, which is expected as the model learns to adjust its parameters to the training set. Red line (validation loss) reflects the error on the validation data, which the model has not seen during training.

The loss trajectories corroborate the model’s balanced performance (mAP@50 = 0.73, precision = 0.75, recall = 0.71). After a short adaptation phase (epochs 1–2), validation loss decreases in parallel with training loss and remains comparable, slightly lower after epoch 4, consistent with effective regularization and adequate data partitioning. In our results, both curves decline and remain close to each other, suggesting the model does not yet exhibit overfitting; rather, training appears stable. Training loss (blue) decreases monotonically from 2.0 to 1.14 across nine epochs, while validation loss (red) shows a brief rise at epoch 2 (1.90) followed by a steady decline to 1.05. The co-decreasing curves, their small final gap (~0.09), and the absence of persistent divergence indicate stable learning with no evidence of overfitting (Figure 5).

**Figure 6:**
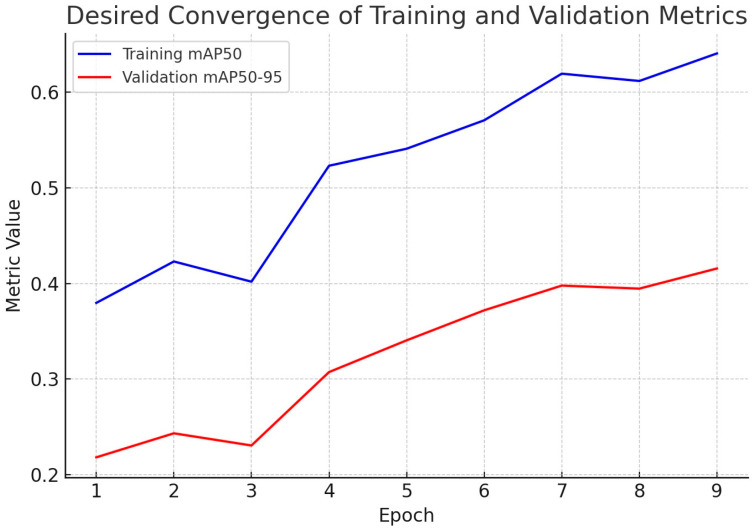
Convergence of detection performance during YOLOv7 training and evolution across nine epochs for the bovine sperm morphology detector. Blue line (training mAP50) indicates the mAP@50 (mean average precision at intersection over union [IoU] = 0.5) on the training set. It describes how well the model predicts on the training data. Red line (validation mAP50–95) shows the mAP@50–95 (stricter, averaging across multiple IoU thresholds) on the validation set.

After a brief adjustment at epoch 3, both curves rise steadily, with training mAP@50 increasing from 0.38 to 0.64 and validation mAP@50–95 from 0.22 to 0.42 by epoch 9. This behavior reflects desirable convergence; the model improves progressively while maintaining coherence between what it learns and how well it generalizes. The gap between curves is expected because mAP@50–95 is a stricter metric than mAP@50; nevertheless, the parallel upward trends and reduced oscillations from epochs 5–9 indicate stable optimization and no evidence of overfitting. These learning dynamics are consistent with the final test set performance (mAP@50 = 0.73, precision = 0.75, recall = 0.71), supporting that the trained YOLOv7 model achieves a balanced tradeoff between accuracy and generalizability for automated assessment of bovine sperm morphology (Figure 6).

**Figure 7:**
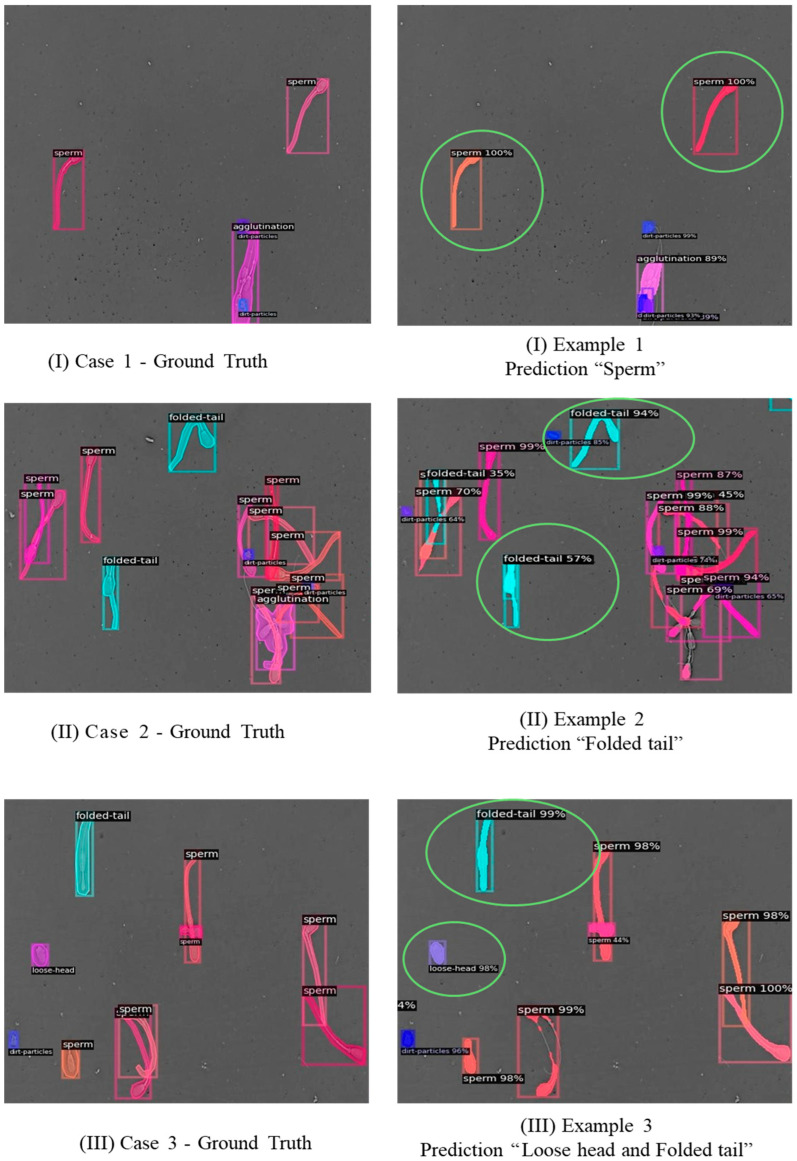
Qualitative comparisons of ground truth and model predictions across three representative examples ((**I**)–(**III**)). Figure on the left indicates ground truth and figure on the right indicates that which is predicted by the deep learning network.

For qualitative examples to visually assess the performance of the proposed model, Figure 7 compares ground truth annotations and model predictions for representative samples. These visualizations illustrate that the model can correctly identify and classify most categories of sperm morphology, although some errors remain in classes with high interclass similarity, such as loose tail (Figure 7).

**Figure 8:**
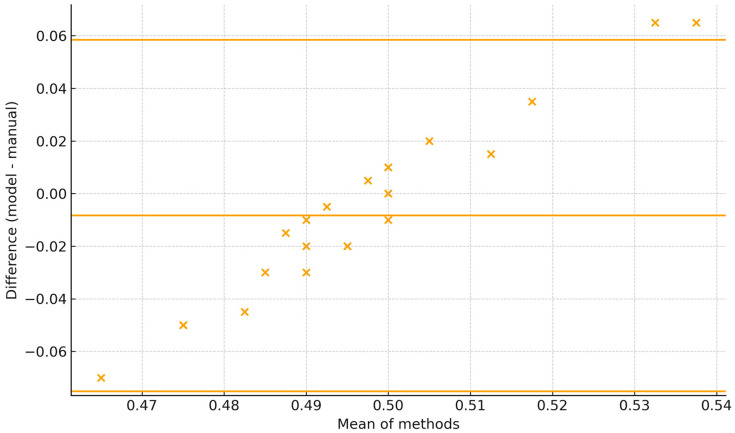
Bland–Altman plot comparing sample level bull sperm morphoanomaly percentages between model and manual sperm morphology assessments.

The Bland–Altman plot shows the 95% limits of agreement span roughly −0.07 to +0.06 (±6–7 percentage points), with all observations falling within these limits. Dispersion is fairly constant across the range with no marked heteroscedasticity; differences tend to be slightly more negative at lower means and positive at higher ones. For BBSE reporting, the current agreement is close to acceptable if a tolerance of ±5 percentage points is used (Figure 8).

## 4. Discussion

The results of this study underscored the variability among six classes of sperm morphology. This disparity is consistent with the visual analysis of some morphological abnormalities, which complicates class separability in automated detection systems [34]. This behavior aligns with the effectiveness of Adam optimization and data augmentation strategies that stabilize training in computer vision tasks [36,37]. The smooth reduction in loss further supports the adequacy of selected hyperparameters.

The findings suggest that the system is sensitive to imaging noise and structural ambiguities, consistent with challenges reported by related work on sperm morphology detection [38,39], and in the case of this study, it may be related to the intrinsic challenge of achieving high sensitivity in datasets with imbalanced representation between classes. The most frequent errors were in the loose tail category, where the model confused these instances with normal spermatozoa or folded tail. This confusion can be attributed to visual overlap in tail morphology and to a limited representation of rare defects in the training set. In terms of detection quality, false positives were primarily associated with background particles or debris misclassified as morphological abnormalities, while false negatives reflected missed detections of subtle tail deformations. Future improvements may involve incorporating domain-specific enhancements to simulate tail deformations or applying attention-based modules to improve the model’s ability to focus on fine-grained morphological cues.

The proposed model performance merits a comparison with recent studies that report detection or classification performance for sperm morphology or sperm cell detection in human or bovine samples. One study analyzed human sperm morphology using mAP (YOLOv5) and obtained a value of 0.731 [39]. Other studies that examined human sperm morphology used mAP (YOLOv5) and the value was 0.7215 [38] or used F0.5i [25], but did not report the obtained value; while the proposed systems analyzed bovine sperm morphology, the present study used mAP@50 and obtained a value of 0.73. Due to differences in reported evaluation protocols (e.g., mAP, F0.5, dataset scope), direct comparison remains limited. The proposed model, however, achieves performance levels comparable to existing YOLO-based approaches in human sperm detection tasks. This suggests that the system’s performance is positioned within the current range of bull sperm morphology detection accuracy.

If ROC-AUC or PR-AUC results become available, they should be included in future comparisons to provide a more comprehensive evaluation of detection thresholds and class imbalances [40]. These comparisons are constrained by the metrics reported in each study and the specific tasks addressed. Direct comparisons are limited; for example, some methods focus on detection (mAP), while others report classification accuracy. Our results, nevertheless, indicate that the proposed system achieves performance in line with recent YOLO-based bull sperm morphology detection frameworks.

### 4.1. Advanced CNN Techniques for Sperm Morphology Analysis

For bovine sperm morphology assessment, unpublished results demonstrated the effectiveness of YOLOv7 in achieving detection and classification accuracies up to 94%, significantly reducing analysis time from approximately 20 min per sample to less than 30 s. Other work further addressed challenges such as small dataset sizes and class imbalance by proposing two deep learning methodologies: deep transfer learning, which fine-tunes a pre-trained VGG19 network, and deep multi-task transfer learning, which integrates transfer and multi-task learning strategies. Their methods improved classification performance, reaching accuracies of 84% (head abnormalities), 94% (vacuoles), and 81% (acrosomes), despite limited data availability [41]. The limitation of small datasets was also present in our model, and even so, accuracies between 0.68 and 0.81 were achieved.

Recently, another study proposed a less supervised learning approach utilizing knowledge distillation (KD) to handle limited labeled data and poor image quality [42], e.g., sperm samples with diluents that generate lumps. Such method employs a pre-trained teacher network to guide a smaller student network trained exclusively on normal sperm images, thus effectively distinguishing abnormalities without direct training on anomalous samples. They reported ROC/AUC scores of 70.4%, 87.6%, and 71.1% for head, vacuole, and acrosome defects, respectively. These results support deploying compact models in resource-limited clinical environments.

The introduction of a sequential deep neural network specifically tailored to target morphological defects in the sperm head, acrosome, and vacuoles, performed well when analyzing unstained, low-resolution images, achieving accuracies of 90%, 89%, and 92% for head, acrosome, and vacuole abnormalities, respectively [32]. This proposed method optimized for real-time applications, significantly reduced computational requirements compared to conventional approaches, which could practically diagnose sperm morphology in both clinical and laboratory contexts.

Recent research has combined morphological and motility analyses to assess sperm quality. It proposed the motion-flow approach, using color-coded HSV representations combined with 3D and 2D CNN models to enhance the simultaneous analysis of sperm morphology and motility. This integrated methodology significantly reduced errors compared to traditional optical flow approaches [13]. Other works have built an ensemble of six CNNs for automated sperm morphology, fusing outputs via soft and hard voting with high accuracies [43]. Relative to YOLO-based human sperm detectors reporting mAP in the 0.72–0.73 band, our bovine model achieved mAP@50 = 0.73 with balanced Precision and Recall, indicating comparable detection despite domain and staining differences. Our main failure mode (loose tail) aligns with slender-structure challenges noted in prior work and motivates attention guided refinements and targeted augmentation for detached tails.

### 4.2. Image Processing Techniques for Sperm Morphology Analysis

Traditional image processing techniques remain crucial, especially during data preparation to enhance quality and reliability of AI-driven analyses. Commonly employed methods include edge detection, noise reduction, and morphological operations to isolate sperm structures such as heads, tails, and cytoplasmic droplets. In bovine contexts, our results described preprocessing techniques to prepare microscopy images for CNN training. These methods typically require manual tuning, nevertheless, and are limited in scalability, prompting increased integration with automated learning-based approaches.

One study recently tested a method that combines image-based flow cytometry (IBFC) with CNNs for automated boar sperm morphology analysis [44]. By utilizing IBFC, the authors obtained high-resolution images of individual sperm cells, simplifying preprocessing and classification of several morphological abnormalities, such as proximal and distal cytoplasmic droplets, distal midpiece reflexes, and coiled tails. Their approach achieved F1 scores up to 99.31%.

Spatially fusing multiple color spaces (RGB, HSV, LAB, YCbCr) with the EfficientNetB3 model on the HuSHeM dataset yielded slightly higher sperm image classification accuracy (74.32%) than any single space (RGB 73.16%, HSV 72.92%, LAB 73.64%, YCbCr 69.2%). This indicates that multicolor space integration can modestly improve reliability in automated sperm morphology analysis [45]. Further expanding on adaptive preprocessing techniques, a structured three stage, adaptive pipeline for detecting abnormalities in sperm cell images was introduced. It showed that orderly preprocessing to handle noise and heterogeneity can improve downstream classification reliability [46].

GAN-based augmentation with a transfer-learning approach (i.e., Transfer-GAN) fine-tuned BigGAN to generate realistic spermatozoa images, thereby alleviating data scarcity and imbalance. It improves morphology classification accuracy of head (84.66%), vacuoles (94.33%), and acrosomes (79.33%), reaching 95.1% on HuSHeM [41]

### 4.3. Technological Tools and Data Acquisition Systems

The Hi-LabSpermMorpho dataset, a large-scale expert-labeled dataset specifically developed for deep learning-based sperm morphology analysis, comprises 49,345 RGB images categorized into 18 detailed abnormality classes covering sperm head, neck, and tail anomalies. These were obtained through three Diff-Quick staining methods (BesLab, Histoplus, and GBL). Extensive benchmarking with 35 deep learning architectures, including CNNs and vision transformers (ViTs), showed consistent classification accuracies ranging between 63.58% and 67.42%, depending on the method. The simplified four-category version of the dataset (head, neck, tail, and normal), moreover, enabled significantly higher accuracy up to 84.20%. Such comprehensive and detailed datasets are valuable resources for training robust deep learning models, overcoming sperm morphology research limitations commonly faced due to dataset size, labeling specificity, and image quality [47]. In contrast, our model has been trained with images taken from samples fixed by pressure and temperature, without the need for staining, and has shown greater accuracy, which could represent an advantage at the methodological level and input requirement in comparative terms.

### 4.4. Challenges and Future Directions in Automated Sperm Morphology Analysis

Research limitations associated with conventional sperm morphology evaluation include significant inter-observer variability and subjectivity inherent in manual inspection [48]. The limitations of traditional staining techniques, such as Diff-Quick, can also produce morphological artifacts and render cells unusable for subsequent treatments [49,50]. To overcome these challenges, an ensemble deep learning model designed specifically for classifying live, unstained human sperm based on whole-cell morphology (head, midpiece, and tail) has emerged. Its approach achieved accuracy and precision of approximately 94% compared to expert consensus and demonstrated consistent performance even when image resolution was significantly lower [48]. This image-based, stain-free approach to sperm morphology analysis could be advantageous, as devices exist that use pressure and temperature to fix sperm samples and capture morphology images without a staining medium [12,30].

Segmentation challenges associated with slender sperm structures, such as the axial filament and twisted tails, continue to haunt the differentiation of closely related morphological features [51]. Both conventional and deep learning segmentation suffer under- or over-segmentation due to structural thinness and midpiece tail similarity, while hybrid pipelines (CNN external and classical internal) add substantial computational cost. Despite advances, efficient and precise algorithms for these slender structures remain a necessity.

Future efforts may focus on strategies to improve classification in minority or visually similar categories, such as loose tail and loose head. Potential directions include the incorporation of larger and more diverse training datasets, advanced augmentation techniques, and class rebalancing methods to mitigate detection bias. In addition, hybrid approaches combining YOLO-based detection with specialized postprocessing (e.g., keypoint-based or attention-guided refinement) may improve discrimination of subtle morphological defects. The exploration of transformer-based detection frameworks and self-supervised pre-training could further generalize data limited scenarios.

Automated morphology can operationalize bovine sperm morphology analysis standardization analogous to WHO human frameworks, the predefined categories quality control, and report templates for Breeding soundness evaluation. In practice, the sperm morphology categories inform compensable vs. uncompensable defect profiles, aiding sire selection, culling decisions, and extender or protocol quality assurance. Publishing open model weights and datasets further enables inter-lab reproducibility and external quality control. Finally, future studies should evaluate the system under extended laboratory conditions, including datasets acquired with different imaging settings and across multiple farms or clinics, to assess robustness, reproducibility, and potential for deployment to veterinary diagnostic and breeding programs.

### 4.5. Limitations

The scope of this study is limited by several factors. First, the dataset is restricted to bovine sperm collected under controlled laboratory conditions from sexually mature, healthy Brahman bulls 2–3 years old with known fertility. These specific characteristics could reduce generalizability to other species or to field environments with variable image quality. Second, the annotations were performed manually, introducing a potential bias that could affect reproducibility. Third, rare categories such as loose tail and loose head are underrepresented, which limits the model to robustly learn their morphological patterns. These limitations indicate that generalizability to other species, such as ovine or human sperm, remains uncertain without additional domain adaptation or transfer learning experiments. Reported results provide evidence, however, that the proposed pipeline can serve as foundational for further research to adapt automated morphology analysis to broader reproductive and clinical contexts.

## 5. Conclusions

This study evaluated the performance of YOLOv7 for the automatic classification of bovine sperm morphology across six categories: normal, agglutination, dirt particles, folded tail, loose head, and loose tail. The results indicate that the model achieved balanced detection performance, with global metrics of mAP@50 = 0.73, precision = 0.75, recall = 0.71, and F1 score = 0.73. Class level analysis revealed variability in detection accuracy, with well-structured categories (normal, folded tail) outperforming fragmented or visually ambiguous classes (loose tail).

From a computational perspective, the model demonstrated real-time inference capacity on GPU hardware and stable convergence across training epochs, with no evidence of severe overfitting. These findings suggest that YOLOv7 provides a technically feasible solution for integration into automated laboratory analysis pipelines.

## Figures and Tables

**Table 1:** Summary of quantitative evaluation results across metrics.

Metric	Best Class	Lowest Class	Global Value
mAP@50	Normal (0.80)	Loose tail (0.67)	0.73
Precision	Normal (0.81)	Loose tail (0.68)	0.75
Recall	Normal (0.78)	Loose tail (0.62)	0.71
F1 score	Folded tail (0.77)	Loose tail (0.65)	0.73

**Table 2:** Class-wise evaluation results for sperm morphology.

Class	Precision	Recall
Normal	0.81	0.78
Agglutination	0.76	0.69
Dirt particles	0.74	0.72
Folded tail	0.79	0.75
Loose head	0.73	0.70
Loose tail	0.68	0.62

## Data Availability

The raw data supporting the conclusions will be made available by the authors, without undue reservation. The executable files, the full dataset of images of bull sperm morphology abnormalities is available in project on Roboflow Universe deposited in https://universe.roboflow.com/boarspermmorphology/bovine-sperm-cells-test, and the trained YOLO model can be found in the following repository (accessed on 10 September 2025).
